# Induction of osteogenic differentiation of MSCs by GSK3β knockdown through GSK3β siRNAs transfection

**DOI:** 10.1371/journal.pone.0355286

**Published:** 2026-08-07

**Authors:** Elena V. Galitsyna, Anastasiia A. Buianova, Tatiana B. Bukharova, Irina A. Krivosheeva, Mikhail Yu. Skoblov, Dmitriy V. Goldshtein

**Affiliations:** 1 Stem Cell Genetics Laboratory, Research Centre for Medical Genetics, Moscow, Russia; 2 Department of Functional Genomics, Research Centre for Medical Genetics, Moscow, Russia; TotiCell Limited, Bangladesh, BANGLADESH

## Abstract

The development of effective strategies for treating bone defects can be based on gene therapy methods aimed at regulating the differentiation of osteoprogenitor cells. One of the approaches is to use siRNA molecules in knockdown systems for genes inhibiting osteogenic cell differentiation. In this work, we aimed at developingapproaches to induce osteogenic differentiation of mesenchymal stem cells (MSCs) by siRNAs-mediated knockdown of GSK3β siRNAs in cultures of MSCs derived from human adipose tissue (AD-MSCs). For this purpose, we compared the transfection efficacy of lipoplexes and polyplexes formed with one of four siRNA molecules and five commercial transfection agents most commonly used in laboratory practice. The most effective transfection agent was found to be linear polyethylenimine (PEI) which demonstrated high cytocompatibility both in free form and in polyplexes (even when maximum concentrations were used). Using the polyplexes formed by the newly designed siRNA and PEI, we constructed a highly efficient *GSK3β* gene knockdown system, which showed effectiveness in AD-MSC cultures. As a result, we demonstrated the osteoinductive properties of *GSK3β* siRNA molecules in these cultures. These results provide a methodological basis for future siRNA-based strategies targeting GSK3β in osteogenic applications, with *in vivo* studies needed to establish its translational potential.

## Introduction

The use of small interfering RNA (siRNA) molecules for organotypic bone tissue regeneration represents a promising approach in regenerative medicine. Mesenchymal stem cells (MSCs) are among the principal in vitro models for the development of gene–cell technologies due to their accessibility from different tissues and their high proliferative and multilineage differentiation potential [1]. Osteogenic differentiation of mammalian stem cells can be induced by classical osteoinductive factors – including recombinant proteins of the BMP and WNT families, dexamethasone, β-glycerophosphate, calcium-containing polymers, L-ascorbic acid and vitamin D metabolites [2–6] – as well as by suppression of inhibitory pathways using small molecules or RNA interference approaches [7–9]. However, many pharmacological and biological osteoinducers act on multiple signaling pathways and may produce pleiotropic effects. RNA interference provides a sequence-directed strategy for post-transcriptional gene suppression and therefore offers greater molecular specificity compared with conventional modulators [10–12].

Despite its sequence specificity, the biological outcome of siRNA experiments critically depends on the delivery conditions. Unmodified siRNA molecules, due to their polyanionic and hydrophilic nature, cannot passively traverse the plasma membrane, are rapidly degraded by nucleases, and may activate innate immune sensing pathways. These well-recognized delivery barriers have stimulated the development of chemical modification strategies and carrier-based systems aimed at improving stability and cellular uptake [13,14]. Although numerous delivery systems have been developed, none of them fully satisfies the criteria of an ideal vector combining high efficiency and minimal cytotoxicity [15,16]. Importantly, siRNA complexes and transfection reagents themselves may induce cell type and reagent-specific inflammatory and cytotoxic responses independently of knockdown target. For example, Yoo et al. [17] demonstrated that inflammatory cytokine induction by siRNAs depends on both cell type and delivery reagent. Similarly, Yang et al. [18] showed that RNAi-mediated effects on viability, cell cycle progression and inflammatory signaling vary substantially depending on the combination of cell type and transfection reagent used in human primary mesenchymal cells. These findings highlight the necessity of a systematic evaluation of delivery systems when studying functional outcomes in MSCs.

**Fig 1:**
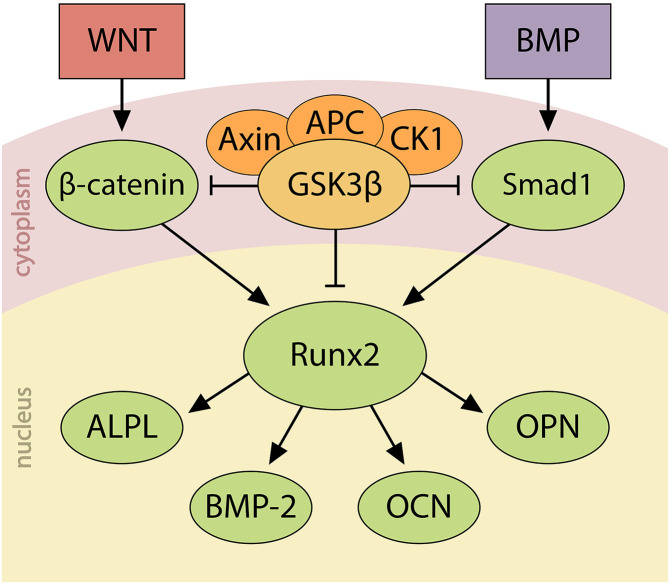
Scheme of molecular interactions between GSK3β, Smad1, and β-catenin during osteogenic differentiation.

The canonical BMP/Smad and WNT/β-catenin signaling pathways are central regulators of osteogenic differentiation and converge on activation of the master transcription factor RUNX2 [19]. Glycogen synthase kinase-3 (GSK3) is a ubiquitously expressed serine/threonine kinase with two highly homologous isoforms, α and β [20,21], originally identified as a regulator of glycogen metabolism [22]. GSK3β as the main player of a multiprotein complex consisting of Axin, APC, CK1 negatively regulates osteogenic signaling by phosphorylating Smad1 and β-catenin, thereby promoting their ubiquitination and proteasomal degradation and limiting transcriptional activation of *RUNX2*-dependent genes [23–25] (Fig 1). In addition, GSK3β modulates Hedgehog and TGF-β signaling pathways relevant to osteogenesis [20,21,26–28].

Genetic and pharmacological studies support the importance of GSK3β in skeletal biology. Dose-dependent phenotypes have been described in murine *Gsk3β* models, and pharmacological inhibition of *GSK3β*, including lithium administration, has been shown to enhance bone formation and regeneration in preclinical models [25,29,30]. However, reported effects of GSK3β modulation on osteogenesis are context-dependent. For instance, Huh et al. [31] reported that *GSK3β* silencing attenuated osteogenic differentiation in murine adipose-derived stromal cells, whereas other studies in human MSCs demonstrated enhanced osteogenic differentiation following *GSK3β* knockdown or inhibition [7–9].

In parallel, the field of RNA interference has shifted towards overcoming major translational barriers related to efficacy, endosomal escape and safety, collectively described as the “three E” challenges of siRNA therapeutics [32]. While advanced delivery platforms, including mesoporous bioactive nanocarriers, have been explored in bone-related applications [33], a comparative benchmarking of widely used commercial transfection reagents in primary human AD-MSCs under osteogenic induction conditions remains limited.

**Fig 2:**
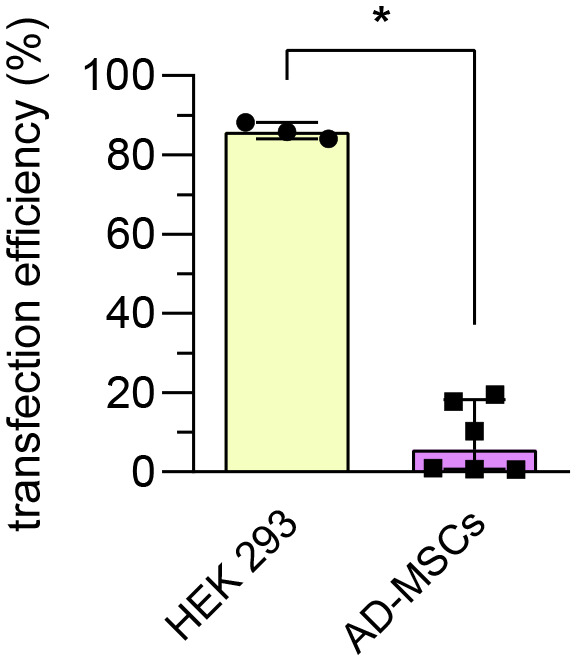
Efficiency of transfection with lipoplexes generated by 75 pmol/mL SC-siRNA and METAFECTENE® PRO transfection agent (1:2) in the HEK 293 cell line and human AD-MSC cultures. Significance of statistical differences for the experimental groups relative to controls at *p* < 0.05 is designated as “*”. Specific data are available in Fig S1.

Therefore, in this work we performed a systematic comparative assessment of lipoplex- and polyplex-based siRNA delivery using four candidate GSK3β-targeting sequences and five commonly used commercial transfection reagents in HEK 293 cells and primary human adipose-derived MSCs. We evaluated knockdown efficiency, cytotoxicity and osteogenic readouts following GSK3β suppression. Additionally, we developed a sequence selection strategy (siRNAfit software) to identify candidate siRNA molecules with improved predicted efficacy. It should be noted that our aim was not to redefine the mechanistic role of *GSK3β* in osteogenesis, but rather to provide a quantitative and methodological framework for optimizing siRNA-mediated gene silencing in primary human AD-MSCs.

## Materials and methods

### Cell cultures

The HEK 293 cell line, primary human adipose tissue-derived MSCs (AD-MSCs), and primary MSCs derived from human exfoliated deciduous teeth (SHED) cultures at passages 2–3 were used in this study. MSC cultures were obtained from tissues of healthy donors recruited between 18 December 2020 and 19 April 2022. All donors provided written informed consent prior to tissue collection. In the case of minors, written informed consent was obtained from their parents or legal guardians. The study was approved by the local Ethics Committee of the Research Centre for Medical Genetics (RCMG), Moscow, Russia (Protocol No. 8/3 dated 14.12.2020). The MSC cultures were confirmed to possess the ability to differentiate into adipogenic, chondrogenic and osteogenic lineages, as well as clonogenicity and immunophenotype verified based on the expression of positive and negative cell CD-surface markers.

Before the experiments, HEK 293 line cells and MSC cultures were cultured in DMEM medium (PanEco, Moscow, Russia) containing 10% FBS (PAA Laboratories, Dartmouth, MA, USA), 4 mM *L*-glutamine (PanEco, Moscow, Russia), 100 mg/L amikacin (Synthesis, Saint Petersburg, Russia), 10 µg/L FGF-2 (ProSpec, Saint Louis, MI, USA) and 2000 IU/L heparin sodium (B. Braun Medical Inc., Melsungen, Germany) under standard conditions: 37 °C and 5% CO_2_. The medium was replaced every 3 days.

### siRNA design

In order to perform a knockdown of *GSK3β* mRNA, the sequences of 4 siRNA molecules targeting different exons of this gene were chosen. The sequences of GSK3β-1 and GSK3β-2 siRNAs were adopted from the studies of Liang M.H., Chuang D.M. 2006, 2007 [34,35], which demonstrated a 70−80% suppression of *GSK3β* gene expression by in hard-to-transfect rodent cell culture models; the GSK3β-3 and GSK3β-4 siRNA molecules were designed using the custom siRNAfit software [36] developed by the Laboratory of Functional Genomics at the RCMG. The software scans the target mRNA sequence using windows of 19–21 nucleotide and scores each oligonucleotide according to the empirical scoring rules described by Laganà et al. (2015) [37]. Candidate siRNAs were filtered to exclude sequences containing discouraged nucleotide motifs and those with a GC content outside the 30–50% range. The remaining candidates were evaluated using a multi-parameter scoring system based on previously established empirical rules. The scoring comprised the following four components:

1. Positional nucleotide weighting – each position in the siRNA duplex was assigned a score according to a set of empirically derived rules (1 point per satisfied rule).
2. Nucleotide composition – penalties and bonuses were applied as follows: –1 point per cytosine, + 1 point per adenine, and an additional +10 points if the number of adenine or uracil residues at the 3′ end exceeded that at the 5′ end.
3. Short sequence motifs – di-, tri-, and tetranucleotide motifs were evaluated, with predefined favourable and unfavourable combinations contributing ±1 point each.
4. Hairpin secondary structure – a user-adjustable penalty was applied for structures exhibiting any of the following features: a loop length <4 nucleotides, a predicted melting temperature >30°C, or a stem length <4 bp.

For off-target specificity filtering, each candidate underwent a local BLASTN search (BLAST+ 2.13.0) against a reference human transcriptome database (compiled from NCBI RefSeq and Ensembl annotations). Matches with a contiguous stretch of ≥13 nucleotides incurred a penalty of 100 points per off-target hit. As an optional validation step, an online BLAST search was performed against the NCBI nucleotide database.

The final output was a ranked list of candidate siRNAs sorted by total score (higher scores indicating more favourable candidates). The top 100 candidates were provided, with per-sequence details including position in the target mRNA, nucleotide sequence, GC content, individual scoring components, and the number of off-target BLAST hits. All scoring parameters were recorded in the output file to ensure transparency and reproducibility.

As a control we have used a scrambled siRNA (SC-siRNA) labelled with 6-carboxyfluorescein (FAM), which does not target any human gene. This SC-siRNA was obtained from Horova et. al (2013) [38] and served as a negative control.

All siRNA sequences used in this study are listed in Table 1. Synthesis of siRNAs was performed by “DNA-Synthesis LLC” (Moscow, Russia).

**Table 1:** Sequences of siRNA molecules used in the study.

Name	Targeting GSK3β exon	Strand	Sequence (5′ → 3′)
Control siRNA-FAM (SC-siRNA)	–	sense	FAM-AGGUCGAACUACGGGUCAAdTdC
		antisense	FAM-UUGACCCGUAGUUCGACCUdAdG
siRNA GSK3β-1 (siRNA-1)	4	sense	UCUGGUGCUGGACUAUGUUdTdT
		antisense	AACAUAGUCCAGCACCAGAdTdT
siRNA GSK3β-2 (siRNA-2)	10	sense	CUGCCCGACUAACACCACUdTdT
		antisense	AGUGGUGUUAGUCGGGCAGdTdT
siRNA GSK3β-3 (siRNA-3)	8	sense	CCAACAAGGGAGCAAAUCAGAdTdT
		antisense	UCUGAUUUGCUCCCUUGUUGGdTdT
siRNA GSK3β-4 (siRNA-4)	7	sense	GAUCAGUUGGUAGAAAUAAUCdTdT
		antisense	GAUUAUUUCUACCAACUGAUCdTdT

### Transfection of siRNAs and analysis of *GSK3β* gene knockdown efficiency in the HEK 293 cell line

HEK 293 cell line was transfected with lipoplexes containing 75 pmol/mL of GSK3β siRNA molecules either alone or in combinations, or control SC-siRNA and METAFECTENE® PRO (Biontex Laboratories GmbH, München, Germany) at a 1:2 ratio (µg of siRNA: µg or µl of transfection agent) (Table 2) according to the previously developed protocol [39]. Lipoplexes were formed in Dulbecco’s phosphate-buffered saline (DPBS) (PanEco, Moscow, Russia) for 15 min. Transfection was performed in a 24-well plate when cells reached 80% confluency in 1 mL of DMEM medium supplemented with 10% FBS (PAA Laboratories, Dartmouth, MA, USA) for 24 hours. Transfection efficiency was monitored by fluorescence microscopy and flow cytometry on the cells transfected with SC-siRNA.

**Table 2:** Quantities and ratios of siRNA and transfection agent molecules used in the experiment.

Volume of siRNA, µg	0.335	0.67	1	1.34
Amount of siRNA, pmol	25	50	75	100
Volume of DPBS/Opti-MEM for dilution of siRNA, µL	30	30	30	30
Volume of DPBS/Opti-MEM for dilution of transfection agent, µL	40	40	40	40
Amount of transfection agent at a ratio of 1:2, µg or µL	0.67	1.34	2.01	2.68
Amount of transfection agent at a ratio of 1:3, µg or µL	1.005	2.01	3	4.02

The knockdown efficiency of the *GSK3β* gene in HEK 293 cells was analyzed by RT-qPCR according to the method as described in in Section “Evaluation of expression of *GSK3β* gene and genes of osteogenic differentiation marker”. Total cell RNA was isolated by guanidine thiocyanate-phenol-chloroform extraction [40]. Reverse transcription was performed using the ImProm-II™ Reverse Transcription System reagent kit (Promega, Madison, WI, USA) according to the manufacturer’s recommendations. The primer sequences for *GSK3β* mRNA are listed in Section “Evaluation of expression of *GSK3β* gene and genes of osteogenic differentiation marker” and Table 3. Primer sequences for reference genes were adopted from the article [39].

**Table 3:** Sequences of primers specific to the *GSK3β* gene, genes of osteogenic differentiation marker, and reference genes.

Name	Sequence (5′ → 3′)
ACTB forward	CCTGGAACCAGCACAATA
ACTB reverse	GGGCCGGACTAGTCATAC
ALPL forward	CTCGGAAGACACTCTGACCGT
ALPL reverse	TAGTCCACCATGGAGACATTCTCT
BMP-2 forward	ACTACCAGAAACGAGTGGGAA
BMP-2 reverse	GCATCTGTTCTCGGAAAACCT
GAPDH forward	GAAGGTGAAGGTCGGAGTACA
GAPDH reverse	TTCACACCCATGACGAGACAT
GSK3β forward	ACGGCACCCAAATATCAAACT
GSK3β reverse	AGCCAGAGGTGGATTACTTGA
OCN forward	CAGAGTCCAGCAAAGGTGCAG
OCN reverse	CTCCCAGCCATTGATACAGGT
OPN forward	GCCGAGGTGATAGTGTGGTT
OPN reverse	AACGGGGATGGCCTTGTATG
RUNX2 forward	AGTGGACGACAGTCTGACTTT
RUNX2 reverse	GGTGAATGCGGTAAGACTGGT

Fluorescence microscopy experiments were performed in 2 biological and 5 technical replicates. RT-qPCR analysis was performed with 3 technical replicates per group.

### Transfection of siRNA molecules into MSC cultures

To determine the effective concentration of siRNA molecules AD-MSCs were transfected with polyplexes containing 25, 50, 75, or 100 pmol/mL of SC-siRNA and linear polyethylenimine (PEI) with a molecular weight of 25 kDa (23966−1; Polyscience, Warrington, PA, USA) at a 1:3 ratio (μg of siRNA: μg or μL of transfection agent) (Table 2).

In order to choose the optimal transfection agent, the HEK 293 cell line and MSC cultures were transfected with lipoplexes/polyplexes consisting of 50 pmol/μL of SC-siRNA and METAFECTENE® PRO (Biontex Laboratories GmbH, München, Germany), Lipofectamine® 2000, Lipofectamine® 3000 with addition of “P3000”, TurboFect (Thermo Fisher Scientific, Waltham, MA, USA) reagent in the ratio 1:2; or PEI at a ratio of 1:2 or 1:3 according to the manufacturers’ recommendations (Table 2).

Lipoplexes/polyplexes were formed in DPBS or Opti-MEM medium (Thermo Fisher Scientific, MA, USA) for 35–40 min. Transfection was performed in a 24-well culture plate when cells reached 70–80% confluency in 1 mL Opti-MEM medium with 5% FBS (PAA Laboratories, Velizy-Villacoublay, France) for 24 hours.

Cells that were incubated in equivalent amounts of Opti-MEM medium with 5% FBS, and addition of DPBS or Opti-MEM were used as controls.

Digital images of transfected cells at the bottom of the culture plates were captured and analyzed using an inverted microscope AG Axio Observer D1 equipped for fluorescence detection and AxioCam HRc camera, as well as ZEN software (Carl Zeiss Microscopy GmbH, Jena, Germany). The efficiency of cell transfection in suspension was assessed using a CyFlow® Space Flow Cytometer (Partec GmbH, Münster, Germany) and FloMax® Software package (Partec GmbH, Münster, Germany). Composite plots were generated using FlowJo™ Software v10.7.1 (Becton Dickinson Bioscience, San Jose, CA, USA).

The experiment was conducted with 5–8 biological replicates and 1 technical replicate per group.

### Selection of cytocompatible concentration of siRNA molecules and transfection agent in polyplexes

To select the optimal concentration of siRNA molecules and transfection agent in polyplexes, we performed a comparative assessment of their effect on the viability of AD-MSC cultures over 7 days following 24 hours of incubation in 24-well plates in 1 mL of Opti-MEM medium supplemented with 5% FBS.

To evaluate the effect of siRNA molecules on cell viability, cells were incubated with SC-siRNA or siRNAs GSK3β at concentrations of 25, 50, 75, or 100 pmol/μL.

To evaluate the effect of transfection agents on cell viability, cultures were incubated with 0.67 to 2.68 μL/mL TurboFect or 1–4 μg/mL PEI (Table 2), which corresponds to a ratio of 1:2 (μg siRNA: μL TurboFect) and 1:3 (μg siRNA: μg PEI) for formation of polyplexes with concentrations of 25, 50, 75, or 100 pmol/μL of siRNA molecules.

The effect of polyplexes containing 25, 50, 75, or 100 pmol/μL of SC-siRNA and TurboFect or PEI at a ratio of 1:2 and 1:3, respectively, on cell viability was then examined (Table 2).

Immediately after transfection and every third day, the culture media were replaced with DMEM medium (PanEco, Moscow, Russia) supplemented with 10% FBS, 4 mM *L*-glutamine (PanEco, Moscow, Russia), and 100 mg/L amikacin (Synthesis, Saint Petersburg, Russia). Cell viability following exposure to siRNA molecules, transfection agents, and polyplexes was assessed on days 1, 4–5, and 7 after transfection using the MTT-test according to the standard technique [41], which correlates with the number of metabolically active cells in a well of the plate.

### MTT-test

MTT (PanEco, Moscow, Russia) was added to the cell cultures at a concentration of 0.5 mg/mL and incubated for 2.5 h at 37 °C. Formazan crystals were extracted from the cells with 500 μL dimethyl sulfoxide (PanEco, Moscow, Russia) by stirring the suspension on an orbital thermoshaker for 20 minutes. Formazan absorbance was measured on an EnSpire® Multimode Plate Reader (PerkinElmer, Waltham, MA, USA) at 570 nm with background correction at 620 nm.

The experiment was conducted with 4–5 biological replicates and 1 technical replicate per group.

### Assessment of *GSK3β* gene knockdown efficiency and osteoinductive properties of GSK3β siRNA molecules

For AD-MSCs were transfected with polyplexes prepared using 50 pmol/mL of siRNAs GSK3β and PEI in a 1:3 ratio (μg siRNA: μg PEI) according to the procedure described in Section “Finding the effective concentration of siRNA molecules”, to induce *GSK3β* gene knockdown and promote osteogenic differentiation.

The negative controls included AD-MSCs transfected with SC-siRNA AD-MSCs cultured in Opti-MEM medium supplemented with 5% FBS, or exposed to DPBS. Volumes were equivalent to those applied in the experimental groups.

Immediately after transfection and every third day, the cultures media were replaced with DMEM (PanEco, Moscow, Russia) supplemented with 10% FBS, 4 mM *L*-glutamine (PanEco, Moscow, Russia), and 100 mg/L amikacin (Synthesis, Saint Petersburg, Russia).

The transfection results were considered positive when the transfection efficiency with SC-siRNA exceeded 90%. In that case, the expression of the *GSK3β* gene and genes of osteogenic differentiation markers genes was evaluated on days 2 and 7 of the experiment, as well as alkaline phosphatase activity and calcium ion levels.

The fluorescence microscopy experiments were performed with 3 biological replicates and 3 technical replicates. RT-qPCR analysis was performed with 3 technical replicates per group.

### Evaluation of *GSK3β* gene expression and osteogenic differentiation marker genes

Total RNA was isolated from MSCs cultures using RNeasy Plus Mini Kit (Quagen, Hilden, Germany), the synthesis of the first chain of total cDNA on RNA matrix was performed using RevertAid kit (Thermo Scientific, Leipzig, Germany) according to the manufacturers’ recommendations.

RT-qPCR was performed using a CFX96 Touch™ thermal cycler (Bio-Rad, USA) using intercalating SYBR Green I dye (Eurogen, Moscow, Russia) and primers specific to the *GSK3β* gene and genes of osteogenic differentiation marker: *ALPL*, *RUNX2*, *BMP-2*, *OCN*, *OPN*. The mRNA expression level of the analyzed genes was normalized to the average expression values of the *GAPDH* and *ACTB* reference genes (Table 3).

The protocol of RT-qPCR protocol included the following steps: denaturation at 95 °C for 6 min followed by 45 cycles of denaturation at 95 °C for 10 seconds, primer annealing at 60 °C for 15 seconds, elongation at 72 °C for 20 seconds. The specificity of RT-qPCR was confirmed by melting curve analysis (from 70 to 98 °C, with 0.5 °C increments in each cycle) and by PAGE. The obtained expression results of the analyzed genes were normalized to the average expression values of the reference genes *GAPDH* and *ACTB* in the same sample. The relative expression of the gene of interest was calculated by the 2^−ΔΔCt^ method.

### Estimation of alkaline phosphatase activity and calcium ions concentration

The activity of ALPL and calcium ions concentration in cell lysates on day 7 of osteogenic differentiation were determined using reagent kits “Alkaline Phosphatase-New Liquid Form” (Vector-Best, Novosibirsk, Russia) and Calcium Colorimetric Assay Kit (Sigma-Aldrich, St. Louis, MI, USA), respectively, according to the manufacturers’ instructions, on an EnSpire Multimode Plate Reader (PerkinElmer, Waltham, MA, USA).

### Statistical analysis

Data were analyzed using GraphPad Prism 8.00 software (GraphPad, La Jolla, CA, USA). Normality and homogeneity of variances were assessed for all datasets. For comparisons between three or more groups, one-way ANOVA was applied. Post hoc pairwise comparisons were performed using Tukey’s HSD test for the HEK 293 transfection experiment (siRNA-1–4, siRNA-multiplex, SC-siRNA) and for intergroup comparisons of transfection efficiency across cell types and reagents (see Table 4). For selected comparisons, Holm–Šidák correction was applied to adjust for multiple testing. When variance heterogeneity was present, Welch ANOVA with Games–Howell correction was used. Differences with *p* ≤ 0.05 were considered statistically significant. Significance relative to control is indicated as *p ≤ 0.05, **p < 0.01, ***p < 0.001.

**Table 4:** Results of the statistical analysis of intergroup comparisons of transfection efficiency in the HEK 293 cell line and SHED and AD-MSC cultures.

	Overall test (p)	HEK 293 vs SHED (p)	HEK 293 vs AD-MSCs (p)	SHED vs AD-MSCs (p)
MET PRO	0.000079	0.000063	0.0041	0.002
Lip 2000	1.46 x 10^–7^	1.23 x 10^–7^	1.74 x 10^–6^	0.000052
Lip 3000	0.000001	1.6 x 10^–6^	0.000014	0.0082
TurboFect	0.118	0.0201	0.3659	0.0022
PEI 1:2	0.0078 (Welch)	0.0254	0.2383	0.106
PEI 1:3	0.0021	0.0067	0.9998	0.0016

## Results

### siRNA design

To date, there is no uniform algorithm for siRNA sequence design [42]. Freeware programs are based on different approaches (empirical rules, BLAST data, neural networks) and may produce varying results when designing siRNAs even for the same target sequence [43–45]. To develop our own software for designing an effective sequence of siRNA molecules, we took the empirical rules described in the review article [37] summarizing a large amount of experimental data. The siRNAfit software analyzes a given mRNA sequence of 19–21 nucleotides, assigning scores to each oligonucleotide according to empirical rules described by Laganà et al. (2015) [37]. Conformity to a rule (e.g., “A in the third position of the sense strand”) adds a score to that sequence, while nonconformity reduces it. The top-30 siRNAs are then analyzed using BLAST software to find potential off-targets. If an siRNA shows complementarity to a non-target sequence for less than 16 consecutive b.p. it may be selected for synthesis [36]. As a result, two different 21-nucleotide-long siRNA sequences (conventionally designated as siRNA-3 and siRNA-4) targeting exons 7 and 8 of the *GSK3β* gene were designed. Also, two additional 19-nucleotides-long siRNAs (conventionally designated as siRNA-1 and siRNA-2) were selected for use described in the articles [34,35]. According to siRNAfit analysis, the siRNA-3 and siRNA-4 sequences scored the highest, 35 and 37 points, respectively. The siRNA-1 sequence scored 16 points and siRNA-2 scored 9 points.

### Transfection of siRNAs and analysis of *GSK3β* gene knockdown efficiency in the HEK 293 cell line

**Fig 3:**
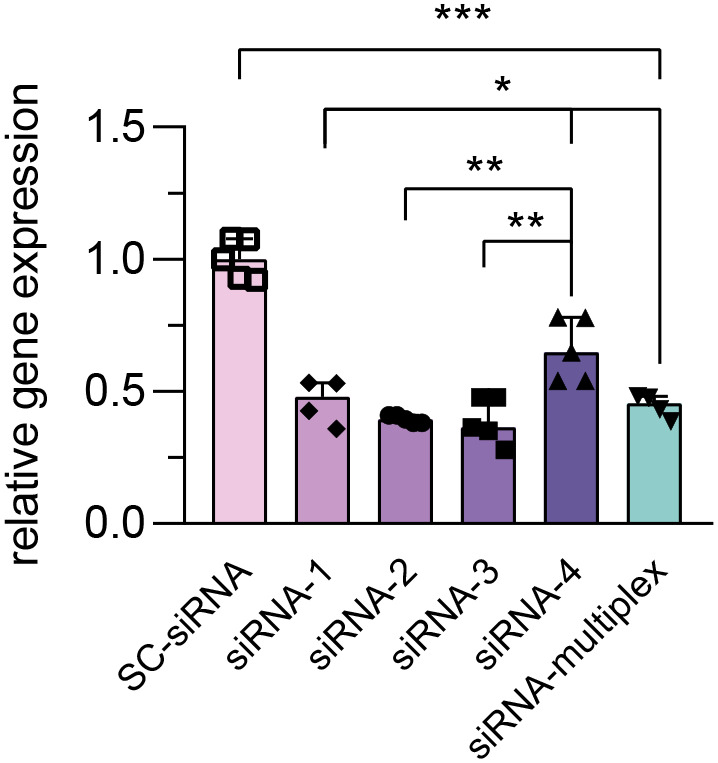
RT-qPCR analysis of *GSK3β* gene expression in the HEK 293 cell line on day 2 after transfection with lipoplexes containing 75 pmol/mL of different GSK3*β* siRNA molecules or a mixture thereof, or SC-siRNA, and METAFECTENE® PRO (1:2). Significance of statistical differences for the experimental groups relative to controls at *p* < 0.05 is designated as “*”, *p* < 0.01—as “**” and *p* < 0.001—as “***”. Full pairwise post-hoc comparisons (Tukey HSD): siRNA-1 vs. siRNA-2, p > 0.05; siRNA-1 vs. siRNA-3, p > 0.05; siRNA-1 vs. siRNA-4, p = 0.0391; siRNA-2 vs. siRNA-3, p > 0.05; siRNA-2 vs. siRNA-4, p = 0.0014; siRNA-3 vs. siRNA-4, p = 0.0011; multiplex vs. siRNA-1, p = 0.9996; multiplex vs. siRNA-2, p = 0.9531; multiplex vs. siRNA-3, p = 0.9273.

To test the efficacy of GSK3β siRNA molecules, we analyzed the *GSK3β* gene knockdown rate on the model cell line HEK 293. The expression analysis of FANTOM5 CAGE promoter-level data revealed that *GSK3β* is actively transcribed in HEK 293 cells, with a dominant promoter exhibiting an RLE-normalized expression value of 12.13 and a cumulative promoter activity of approximately 14.7 across all annotated transcription start sites [46]. In addition, this cell line is characterized by high proliferative activity, which should ensure high efficiency of transfection [47]. To suppress the expression of the target gene, HEK 293 cells were transfected with GSK3β siRNA 1–4 individually as well as with a mixture of all four GSK3β siRNA (siRNA-multiplex). Cells of the control group were transfected with SC-siRNA molecules. The transfection agent METAFECTENE® PRO and a siRNA concentration of 75 pmol/mL were selected to increase the efficiency of siRNA delivery to HEK 293 cells. The transfection efficiency in the experiment equaled to 84% (S1 Fig, Fig 2). 24 hours after transfection with GSK3β siRNA molecules, the expression of the *GSK3β* gene was decreased by 57.05 ± 9.75% in the presence of siRNA-1, by 60.43 ± 1.44% in the presence of siRNA-2, and by 35.21 ± 13.06% in the presence of siRNA-4 relative to its expression in controls (Fig 3). The most effective decrease in *GSK3β* expression was caused by siRNA-3 – by 63.64 ± 11.27%. The use of an equimolar mixture of all four siRNAs was slightly less effective than the use of most siRNA molecules alone and demonstrated the decrease in expression by 56.83 ± 5.06%.

One-way ANOVA revealed a significant effect of siRNA treatment on GSK3β expression in HEK 293 cells (F = 38.81, p < 0.0001). The post-hoc Tukey HSD analysis demonstrated that all individual siRNA molecules and the multiplex combination significantly reduced GSK3β expression compared with the SC-siRNA control (p < 0.001 in all cases), with siRNA-3 producing the strongest knockdown. The multiplex combination achieved a level of suppression comparable to that of siRNA-1, siRNA-2, and siRNA-3, but was significantly less effective than siRNA-4 (p = 0.0193).

### Finding the effective concentration of siRNA molecules

Using flow cytometry and fluorescence microscopy, we determined the effective concentration of siRNA molecules during transfection of AD-MSCs cells.

The METAFECTENE® PRO reagent proved to be ineffective – 20% or less transfected cells in culture (S1 Fig), when optimizing the technique of transfection of MSC cultures, while the PEI transfection agent showed the best result. PEI was used to select the effective concentration of SC-siRNA molecules.

Polyplexes containing 50–100 pmol/mL of SC-siRNA and PEI transfected more than 91.8% of cells in culture, whereas polyplexes containing 25 pmol/mL transfected only 55.1 ± 3.67% of cells.

**Fig 4:**
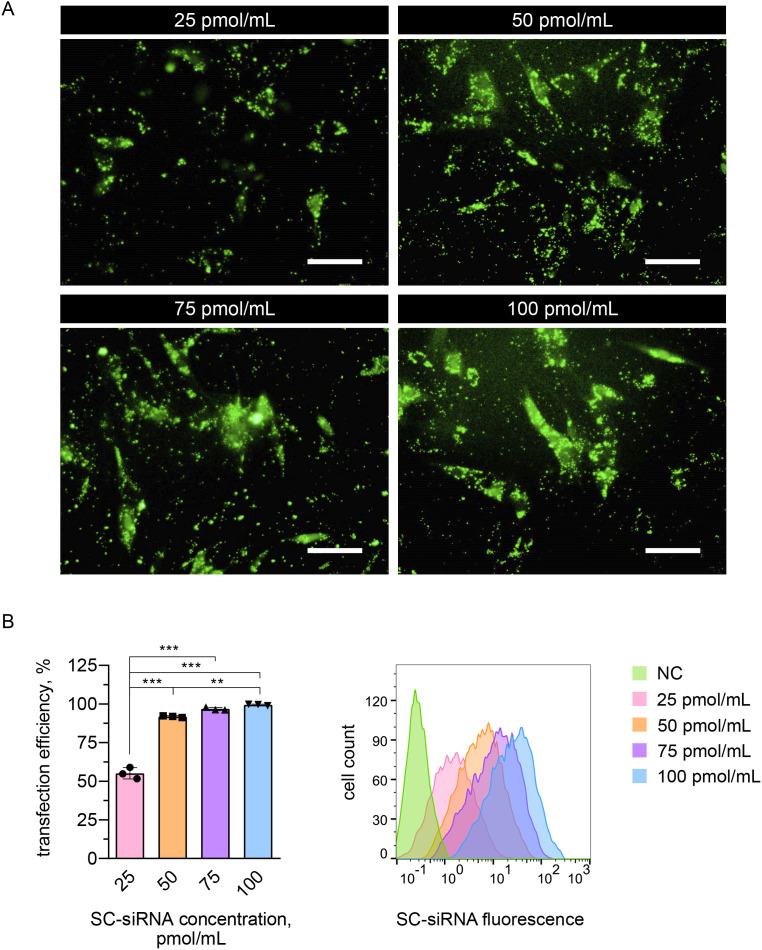
Assessment of transfection efficiency of polyplexes containing scrambled control with 6-FAM fluorescent label (SC-siRNA) and PEI (1:3) by fluorescence microscopy (A) and flow cytometry (B). Scale bars: 100 μm. The number of cells analyzed are listed in Appendix A. NC – negative control. Significance of statistical differences for the experimental groups relative to controls at *p* < 0.01 is designated as “**” and *p* < 0.001—as “***”.

It was shown that there is a direct correlation between the concentrations of siRNA, the transfection agent, and the transfection efficiency of the AD-MSCs cultures (Fig 4). Statistical analysis using one-way ANOVA indicated a highly significant overall effect of the different siRNA treatments on *GSK3β* expression (p = 8.65 × 10^–9^). Post hoc pairwise comparisons using Tukey’s HSD test revealed significant differences between SC-siRNA and all siRNA treatments (25 vs 50 pmol/mL: p < 0.001; 25 vs 75: p < 0.001; 25 vs 100: p < 0.001; 50 vs 100: p = 0.0058), whereas differences between intermediate concentrations were either marginal or non-significant (50 vs 75: p = 0.0539; 75 vs 100: p = 0.3994). These results demonstrate that siRNA-3 provided the most effective knockdown of *GSK3β* in HEK 293 cells under the chosen transfection conditions.

### Evaluation of effects of siRNA molecules on cell viability

**Fig 5:**
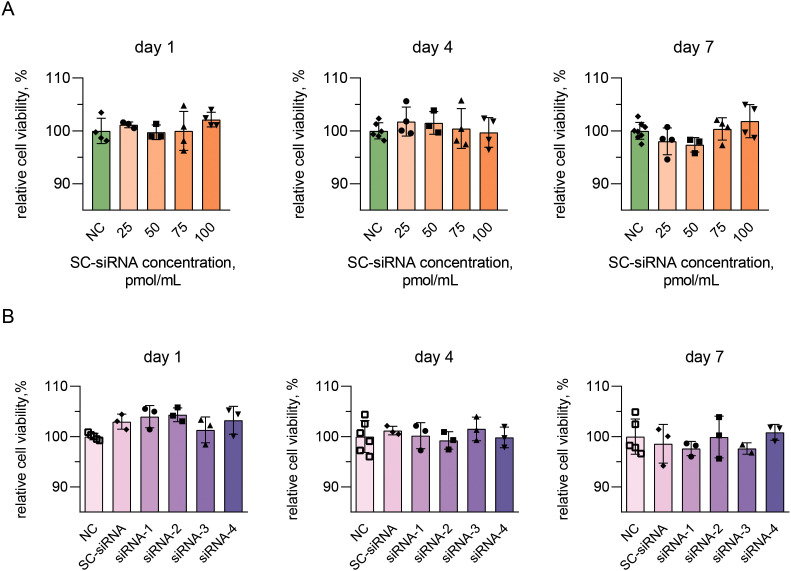
Relative cell viability of AD-MSCs assayed by the MTT-test in the presence of SC-siRNA molecules at concentrations of 25-100 pmol/mL on days 1, 4 and 7 of the experiment (A). Relative cell viability of AD-MSCs assayed by the MTT-test in the presence of GSK3β siRNA molecules at a concentration of 50 pmol/mL on days 1, 4 and 7 of the experiment (**B**). NC – negative control.

When SC-siRNA molecules at concentrations of 25−100 pmol/mL and GSK3β siRNAs at concentrations of 50 pmol/mL were used for transfection, viability of the AD-MSCs at the end of the experiment (7 day) was at least 97.6% of the control group values. No statistically significant differences were found between the experimental and control groups, indicating no measurable effect of SC-siRNA and siRNAs GSK3β-1–4 on cell viability at the selected concentrations (Fig 5A, 5B).

### Evaluation of the effect of the PEI transfection agent on cell viability

**Fig 6:**
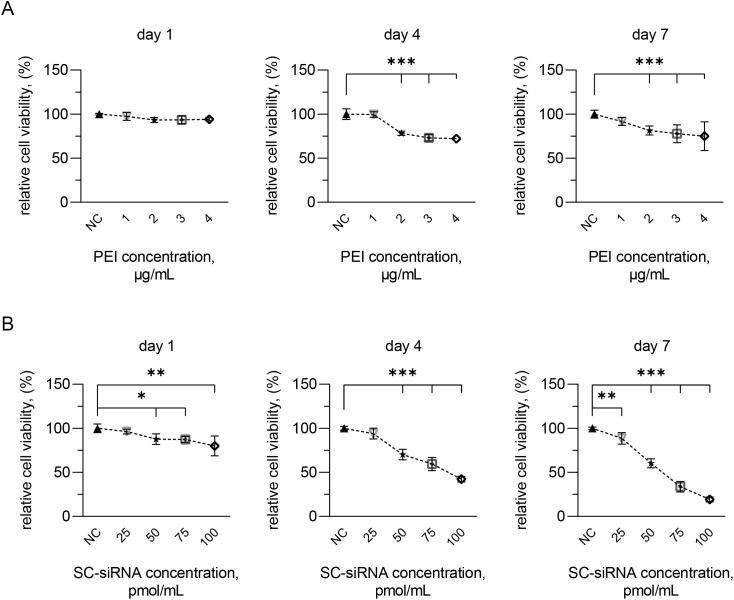
Relative cell viability of AD-MSCs assayed by the MTT-test in the presence of 1-4 µg PEI (corresponding to 25, 50, 75 and 100 pmol/mL siRNA at a 1:3 ratio) on days 1, 4 and 7 of the experiment (A). Relative cell viability of AD-MSCs assayed by the MTT-test in the presence of polyplexes formed with 25-100 pmol/mL of SC-siRNA molecules and 2 μg PEI at a 1:3 ratio on days 1, 4, and 7 after transfection (**B**). NC – negative control. Significance of statistical differences for the experimental groups relative to controls at p ≤ 0.05 is designated as “*”, p < 0.01—as “**” and p < 0.001—as “***”.

On the 1st day of the experiment the viability of the AD-MSCs in all experimental groups was more than 93.4% relative to the values of the control group. No statistically significant differences were found between the experimental and control groups (Fig 6).

The greatest reduction in cell viability was observed on days 4 and 7 of the experiment using the maximum concentrations of the transfection agent – 3 and 4 µg/mL. Cell viability relative to the control group was at least 72.3% and 75%, respectively. According to the international standard ISO 10993−5 [48], cell viability above 70% relative to the negative control indicates good biocompatibility. The use of lower concentrations (1 and 2 µg/mL) of PEI had a smaller effect on cell survival – 81.4% and a higher proportion of viable cells in culture at the end of the experiment (Fig 6A).

Thus, PEI did not cause a marked decrease in cell viability in AD-MSCs cultures at concentrations of 3–4 µg/mL, corresponding to 75–100 pmol/µL of siRNA during polyplexes formation, and demonstrates biocompatibility approximating the control group at concentrations of 1–2 µg/mL, corresponding to 25–50 pmol/mL of siRNA at a 1:3 ratio (µg of siRNA: µg of PEI). An inverse relationship between the concentration of the transfection agent and cell viability is observed.

### Evaluation of the effect of PEI-containing polyplexes on cell viability

On day 1 after transfection, all PEI-formed polyplexes, except those containing 25 pmol/mL SC-siRNA, moderately reduced cell viability relative to the control group by no more than 20%.

On day 4 of the experiment, cell viability in all experimental groups with polyplexes formed with PEI differed statistically significantly from the control (p < 0.0001) and comprised at least 59.4% in the groups of polyplexes with SC-siRNAs concentrations of 25–75 pmol/mL and 42.3 ± 3.2% in the group with a SC-siRNA concentration of 100 pmol/mL. In the presence of polyplexes containing 50 pmol/mL of SC-siRNA and PEI, the relative viability was 70.15 ± 5.94% of the cells in culture.

The greatest reduction in cell viability following the exposure to polyplexes was observed on day 7 in the experimental groups with the highest SC-siRNA and PEI concentrations. In the presence of polyplexes containing 25 pmol/mL and 50 pmol/mL of SC-siRNA and PEI, the relative viability of the cells in culture was 88.6 ± 6.48% and 60.21 ± 5.07%, respectively. Polyplexes containing 75 and 100 pmol/mL of SC-siRNA and PEI showed the lowest cell viability: 33.7 ± 6.02% and 19.26 ± 2.86% of viable cells in culture (Fig 5B).

According to the ISO 10993−5 recommendations [48], polyplexes containing 25 pmol/mL of SC-siRNA demonstrate high biocompatibility when incubated with MSC cultures, and polyplexes containing 50 pmol/mL – satisfactory (close to high) biocompatibility during 7 days of the experiment. Taking into account the transfection efficiency obtained using these concentrations (as described in Section “Finding the effective concentration of siRNA molecules”), the siRNA concentration of 50 pmol/mL was chosen for the following experimental works.

We can conclude that there is an inverse dependence between the siRNA concentration and the PEI transfection agent in polyplexes and cell viability.

In addition, the transfection agent PEI and siRNA molecules when applied separately, exerted a significantly smaller effect on the viability of AD-MSC cultures compared with the polyplexes formed by them.

### Choosing a transfection agent

The efficiency of transfection with lipoplexes/polyplexes formed with 50 pmol/mL of SC-siRNA and various transfection agents in the HEK 293 cell line and AD-MSCs and SHED cultures was studied.

The highest transfection results were achieved in the HEK 293 cell line, including with liposomal agents, which confirms the available observations in the literature [47].

**Fig 7:**
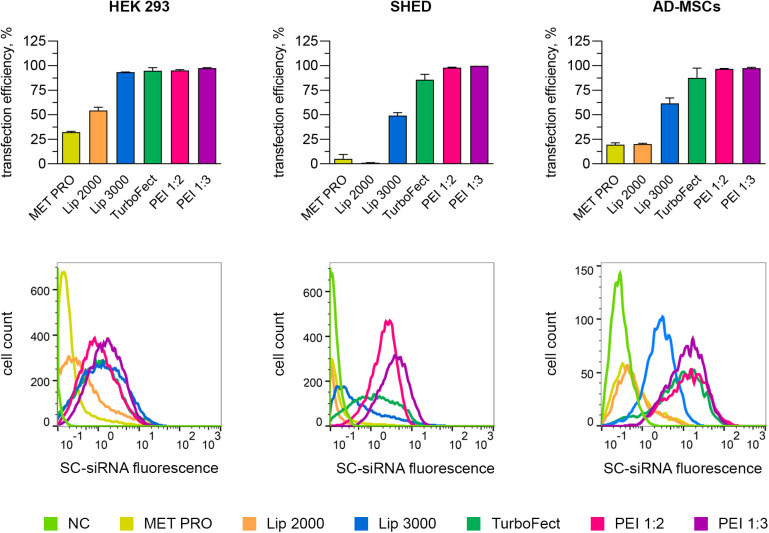
Efficiency of transfection with lipoplexes/polyplexes generated by SC-siRNA and different transfection agents in the HEK 293 cell line and cultures of human AD-MSCs and SHED. Intergroup comparisons were performed separately for each transfection reagent. Homogeneity of variances was assessed using Levene’s test. If assumptions were met, one-way ANOVA followed by Tukey’s post hoc test was applied. In cases of unequal variances, Welch ANOVA with Games–Howell correction was used. MET PRO: METAFECTENE® PRO; Lip 2000: Lipofectamine® 2000; Lip 3000: Lipofectamine® 3000. The amounts of cells analyzed are given in Appendix B. NC – negative control. Specific data are available in Fig S2.

To deliver siRNA molecules into AD-MSCs and SHED, transfection agents based on cationic polymers were more effective than those based on liposomes. The highest transfection efficiency was achieved with the transfection agent PEI – up to 97.5% at a 1:3 ratio and up to 96.8% at a 1:2 ratio of transfected cells in MSC cultures (Fig 7). The efficiency of transfection differed significantly between cell types for most reagents tested (Table 4). Liposome-based reagents (Lip2000 and Lip3000) generally demonstrated highly significant differences between HEK293, SHED, and AD-MSC cultures, although the AD-MSCs vs SHED comparison for Lip3000 was not significant. In contrast, TurboFect did not show a consistent overall effect across cell types (p = 0.118). For PEI 1:2, Welch ANOVA revealed a significant overall effect (p = 0.0078), with HEK293 vs SHED being the only significant pairwise comparison (p = 0.025). PEI 1:3 showed significant differences between SHED and both HEK293 and AD-MSC cells, while HEK293 and AD-MSC did not differ (p = 0.9998). These results indicate that transfection efficiency is reagent- and cell type-dependent, with SHED cells generally being more resistant to transfection.

### Assessment of *GSK3β* gene knockdown and the expression of osteogenic differentiation marker genes in AD-MSC cultures incubated with GSK3β siRNAs

Based on the data described in the literature, the time points 2 and 7 days after transfection were selected to evaluate the knockdown and osteoinductive effects of GSK3β siRNA molecules in MSC cultures [9,49].

On day 2 after transfection with GSK3β siRNA molecules the *GSK3β* gene expression decreased by 20.75 ± 9.94% in the presence of siRNA-1, by 57.21 ± 1.38% in the presence of siRNA-2, and by 50.5 ± 1.96% in the presence of siRNA-4 relative to its expression in the control group. *GSK3β* expression was most effectively reduced by siRNA-3 molecules by 83 ± 1.63%.

**Fig 8:**
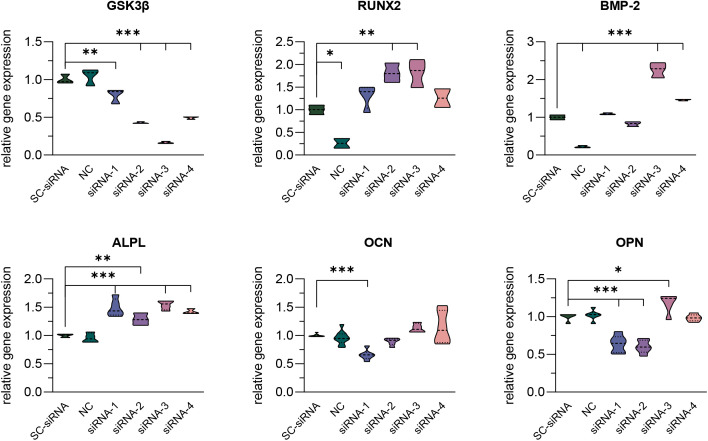
Expression of the *GSK3β* gene and osteogenic differentiation marker genes in AD-MSC cultures on day 2 after transfection with polyplexes containing GSK3β siRNA or SC-siRNA and PEI molecules (1:3) estimated by qRT-PCR. NC – negative control. Significance of statistical differences for the experimental groups relative to controls at p ≤ 0.05 is designated as “*”, p < 0.01—as “**” and p < 0.001—as “***”.

The *RUNX2* gene expression in the presence of each GSK3β siRNA increased 1.3-1.8-fold. Incubation with siRNA-3 molecules also resulted in a statistically significant increase in the expression of the other marker genes (except *OCN*): *ALPL* 1.5 ± 0.1-fold (p < 0.001), *BMP-2* 2.3 ± 0.2-fold (p < 0.001), *OPN* 1.2 ± 0.1-fold (p = 0.01) (Fig 8).

On day 7 after transfection with GSK3β siRNA molecules, the *GSK3β* gene expression returned to baseline levels relative to its expression in the control group. The expression of osteogenic differentiation marker genes in the presence of siRNA-3 molecules increased on average 3-fold compared with the previous time point.

The expression of *RUNX2* gene in the presence of each GSK3β siRNA molecule increased 1.9-3.8-fold in comparison with the control group, the highest increase was observed after incubation with siRNA-3 molecules, reaching 3.8 ± 0.4-fold (p < 0.001).

**Fig 9:**
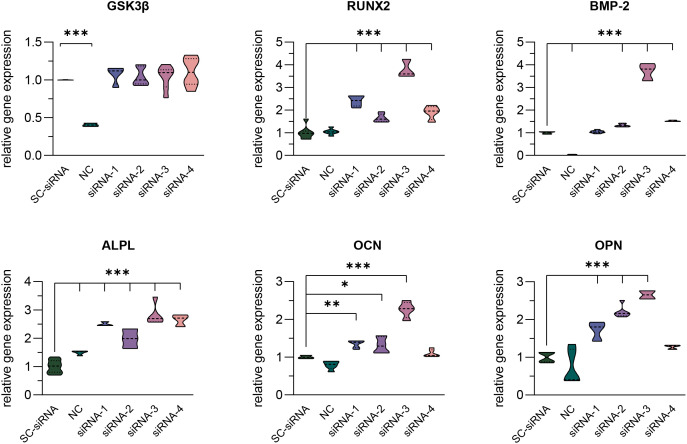
Expression of the *GSK3β* gene and genes of osteogenic differentiation markers in the AD-MSC cultures on day 7 after transfection with polyplexes containing GSK3β siRNA or SC-siRNA and PEI molecules (1:3) estimated by RT-qPCR. NC – negative control. Significance of statistical differences for the experimental groups relative to controls at p ≤ 0.05 is designated as “*”, p < 0.01—as “**” and p < 0.001—as “***”.

Incubation with siRNA-3 also resulted in the highest increase in expression of other marker genes: *ALPL* by 2.8 ± 0.4 times, *BMP-2* by 3.7 ± 0.4 times, *OCN* by 2.3 ± 0.2 times, *OPN* by 2.7 ± 0.2 times (p < 0.001) (Fig 9).

According to the results obtained, the effective knockdown of *GSK3β* gene and osteoinductive effect of GSK3β siRNA molecules at the concentration of 50 pmol/μL in the AD-MSC cultures were shown. GSK3β-3 siRNA molecules have the most marked effect.

### Measurement of alkaline phosphatase activity, calcium ions concentrations and visual assessment of ECM mineralization in AD-MSC cultures after transfection with GSK3β siRNAs

**Fig 10:**
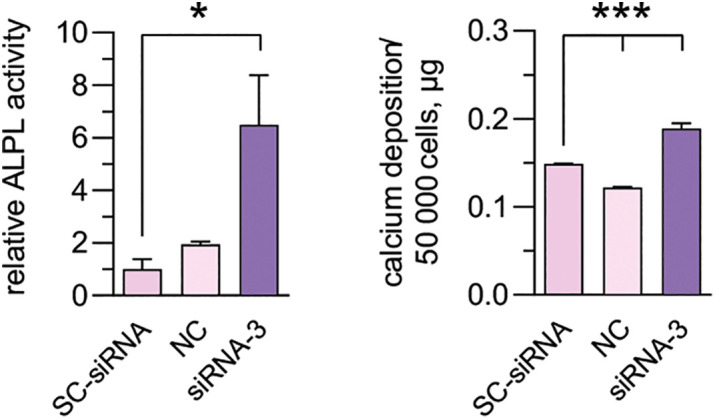
ALPL activity and Ca^2+^ concentration in cell lysates of AD-MSC cultures on day 7 after incubation with polyplexes containing GSK3β siRNAs or SC-siRNA and PEI molecules (1:3) estimated by colorimetric assays. NC – negative control. Significance of statistical differences for the experimental groups relative to controls at *p* < 0.05 is designated as “*” and *p* < 0.001—as “***”.

The transfection of AD-MSC cultures with GSK3β-3 siRNA molecules contributed to a statistically significant increase in ALPL activity and calcium ion concentration (Ca^2+^) by day 7 of the experiment. When cell cultures were incubated with GSK3β-3 siRNAs, ALPL activity and calcium ion concentration in cell lysates increased 6.4 ± 2.3 times (p = 0.05) and 1.3 ± 0.1 times (p < 0.0001), respectively, in comparison with the control group (Fig 10).

**Fig 11:**
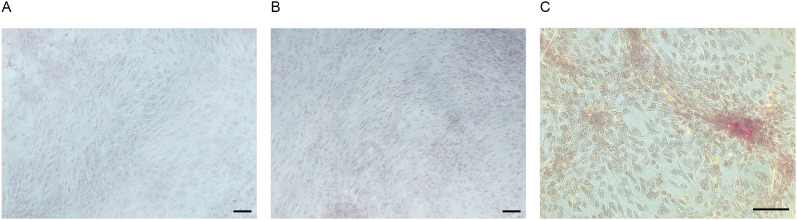
ECM mineralization in AD-MSC cultures on day 7: negative control (NC, A), SC-siRNA (B), and GSK3β siRNAs (C), estimated by colorimetric assays using Alizarin Red staining. Scale bars: 100 μm.

Alizarin Red staining revealed calcified nodules indicative of ECM mineralization on day 7 of MSC cultivation in a medium supplemented with GSK3β siRNA molecules (Fig 11). Its volume exceeded that observed in both SC-siRNA and NC group.

Thus, the positive effect of GSK3β-3 siRNAs transfection on the regulation of osteogenic differentiation of AD-MSC cultures was confirmed.

## Discussion

The present study provides a systematic methodological framework for siRNA-mediated *GSK3β* knockdown in primary AD-MSCs, addressing both siRNA delivery optimization and functional osteogenic outcomes. While the negative regulatory role of GSK3β in osteogenic signaling pathways has been established through pharmacological and genetic approaches [23–25,29,30], the application of siRNA-based *GSK3β* silencing strategies to primary human MSC cultures remains underexplored, with existing reports yielding conflicting results depending on cell source and experimental conditions [7–9,31].

We conducted a comparative evaluation of the efficacy of four siRNA sequences targeting *GSK3β* mRNA. Two siRNA molecules (conventionally designated as siRNA-1 and siRNA-2 in this work), were selected based on prior evidence of their effectiveness in suppressing *GSK3β* mRNA in difficult-to-transfect cell cultures, were chosen from studies of Liang M.H. and Chuang D.M. [34,35]. The remaining, two molecules (conventionally designated as siRNA-3 and siRNA-4 in this work) were selected using siRNAfit software (developed by the authors of the study). In studies by Liang M.H. and Chuang D.M., GSK3β siRNA sequences or their combinations caused a 70–80% reduction in the corresponding protein levels 48 hours after transfection in primary cultures of rat cortical neurons. Transfection was performed using a polyamine-based chemical agent, and *GSK3β* knockdown was confirmed by Western blot analysis [34,35]. The results of the present study, within the same timeframe, showed a reduction of GSK3β expression in HEK 293 cells using siRNA-1 and siRNA-2 by only 57 and 60%, respectively, and in MSC cultures by 21% and 57%, respectively. The most probable reasons for these systematic differences are heterogeneity in cell types, variations in transfection chemistries, and differences in detection methodologies [17,18]. The sequence of siRNA-3, selected using the bioinformatic approach developed in this study, demonstrated more efficient knockdown of the *GSK3β* gene compared to other molecules in both HEK 293 and AD-MSC cultures – 64% and 83%, respectively.

According to data compiled in the siRecords database, which contains information on more than 17,000 siRNA sequences targeting mammalian genes, less than 35% of experimentally tested siRNAs induce more than 90% gene silencing, whereas nearly 20% result in less than 50% efficiency [50,51]. Thus, the design of high-performance siRNA sequences remains imperfect and context-dependent [52].

The relatively limited diversity of early siRNA design algorithms may be explained by the restricted number of sequences used during their development, as well as by their reliance on MPI principles, the Ui-Tei rule, or Reynolds’ rules [52,53]. In this work, the design process was guided by the criteria summarized by Laganà et al. [37]. The siRNAfit algorithm partially overlaps with the Ui-Tei and Reynolds’ rules but does not fully replicate them. In addition, it incorporates filters based on the presence or absence of short 2–4-nucleotide motifs that empirically promote or hinder RNA interference efficiency. Thus, siRNAfit represents an integrative refinement of previously described approaches for the design of 19–21-nucleotide siRNA sequences. Nevertheless, broader validation across additional targets will be required to fully assess its predictive robustness.

Chemical transfection agents may exhibit cytotoxicity depending on their composition and physicochemical properties [54,55]. In the current study, individual administration of siRNAs and PEI exerted no detectable effect on cell viability; however, their formulation into polyplexes resulted in a moderate reduction in cell viability. Similarly, Kafil and Omidi demonstrated that polyplexes formed with linear PEI (25 kDa) and pDNA induced significantly greater cytotoxicity compared to the same transfection agent alone (free PEI) [56]. Godbey et al. reported two distinct sources of cytotoxicity during PEI-mediated transfection: toxicity caused by free dissolved PEI and toxicity associated with intracellular processing of PEI/pDNA polyplexes [57]. Collectively, these data suggest that the increased cytotoxicity of polyplexed linear PEI primarily arises from the intracellular trafficking and processing of these macromolecular complexes.

An inverse relationship between transfection efficiency and cell viability was observed in AD-MSC cultures, consistent with the article of Hoare et al. reporting that increasing concentrations of cationic polymer-based transfection agents enhanced delivery efficiency but significantly reduced viability in human bone marrow–derived MSCs [58]. Given that mesenchymal stem cells are highly sensitive to environmental stressors, such delivery-associated cytotoxicity may represent a biologically relevant confounding factor in differentiation studies, rather than a purely technical limitation.

The role of GSK3β in osteogenic differentiation has been extensively investigated. In the study by Huh et al. the inhibition of *GSK3β* with siRNAs was shown to negatively regulate the osteogenic differentiation of murine AD-MSCs [31]. In contrast, other studies have reported that inhibition of *GSK3β* using siRNA molecules [9], lithium chloride [59], or other small molecules [7,8,60,61] promotes osteogenic differentiation of MSCs. For example, Wang et al. showed that *GSK3β* knockdown in human AD-MSC cultures increased mRNA expression and protein levels of RUNX2, Osterix, SATB2, BSP, OPN, and OCN, enhanced ALPL activity, and elevated calcium deposition, whereas *GSK3β* overexpression inhibited these processes [9]. At the system level, context-dependent effects of WNT signaling in MSC osteogenesis have also been reported, including differential responses depending on MSC origin, as shown by Nantavisai S. et al. [62].

Taken together, these data indicate that the phenotypic consequences of modulating *GSK3β* gene expression are critically dictated by species-specific factors, the anatomical source of MSCs, and cellular signaling pathways. The findings of this study substantiate the premise that under the utilized experimental conditions GSK3β predominantly functions as a negative regulator of osteogenesis in human AD-MSCs. It is important to note that the aim of this study was not to redefine the mechanistic role of GSK3β in osteogenesis. Rather the focus was on evaluating the impact of siRNA delivery efficiency, sequence characteristics, and cytotoxicity of siRNAs, transfection agents, and the polyplexes they form on functional gene silencing in primary human AD-MSCs. It should also be emphasized, that modulation of osteogenic signaling in primary mesenchymal stem cells by siRNAs should be interpreted in the broader context of cellular responses, which are dependent on the drug delivery route.

Finally, it should be acknowledged that the present study was conducted under *in vitro* conditions using commercially available transfection reagents. While translational research increasingly focuses on overcoming *in vivo* delivery challenges, reproducible *in vitro* benchmarking remains essential for mechanistic studies in primary human cells [32]. Future work should incorporate assessment of innate immune activation markers and expanded protein-level pathway analysis to further disentangle delivery-associated effects from sequence-specific gene silencing.

## Conclusions

siRNA molecules, acting via the RNA interference mechanism, represent a highly precise tool for genetic silencing of target mRNA transcripts without directly effecting the genome. However, the chemical transfection vectors utilized for their delivery often exhibit inherent cytotoxicity [52,53]. In the course of this work, we identified PEI as the most effective transfection agent for MSCs and determined the optimal concentration of siRNA molecules in polyplexes to be 50 pmol/mL.

This study demonstrates that while isolated siRNA molecules and transfection agents exert no cytotoxic effects on MMSC cultures, their formulation into polyplexes induces moderate cytotoxicity. Furthermore, we established a distinct dose-dependent profile: the concentrations of siRNA and the transfection agent correlate directly with transfection efficiency, yet exhibit a negative correlation with cell viability. Finally, the pronounced osteoinductive properties of GSK3β siRNA molecules in human MSC cultures were demonstrated. The developed GSK3β-3 siRNA sequence showed the best result of target gene knockdown and osteoinductive effect simultaneously. Due to the ability to positively regulate the osteogenic differentiation of MSCs, this sequence is recommended for research purposes for the induction and enhancement of the osteogenic differentiation of MSCs.

These findings provide a methodological foundation that may inform future preclinical development of siRNA-based approaches for osteogenic induction, although *in vivo* validation will be required before any translational application can be considered.

## Supporting information

S1 Table The number of cells analyzed when evaluating the efficiency of transfection with polyplexes containing control SC-siRNA and PEI (1:3) by flow cytometry. (XLSX)

S2 Table The number of cells analyzed in the evaluation of transfection efficiency of lipoplexes/polyplexes formed with different transfection agents on HEK 293 cells and human AD-MSCs and SHED cultures. (XLSX)

S1 Fig Comparative transfection efficiency of METAFECTENE® PRO lipoplexes in AD-MSC and HEK 293 cell lines. (TIF)

S2 Fig Transfection efficiency of SC-siRNA lipoplexes and polyplexes in HEK 293, AD-MSC, and SHED cultures. (TIF)

## References

[1] Hamann A, Nguyen A, Pannier AK. Nucleic acid delivery to mesenchymal stem cells: a review of nonviral methods and applications. J Biol Eng. 2019;13:7. doi: 10.1186/s13036-019-0140-0 30675180 PMC6339289

[2] Beederman M, Lamplot JD, Nan G, Wang J, Liu X, Yin L, et al. BMP signaling in mesenchymal stem cell differentiation and bone formation. J Biomed Sci Eng. 2013;6(8A):32–52. doi: 10.4236/jbise.2013.68A1004 26819651 PMC4725591

[3] James AW. Review of signaling pathways governing MSC osteogenic and adipogenic differentiation. Scientifica (Cairo). 2013;2013:684736. doi: 10.1155/2013/684736 24416618 PMC3874981

[4] Langenbach F, Handschel J. Effects of dexamethasone, ascorbic acid and β-glycerophosphate on the osteogenic differentiation of stem cells in vitro. Stem Cell Res Ther. 2013;4(5):117. doi: 10.1186/scrt328 24073831 PMC3854789

[5] Nifant’ev I, Bukharova T, Dyakonov A, Goldshtein D, Galitsyna E, Kosarev M, et al. Osteogenic differentiation of human adipose tissue-derived MSCs by non-toxic calcium poly(ethylene phosphate)s. Int J Mol Sci. 2019;20(24):6242. doi: 10.3390/ijms20246242 31835689 PMC6940807

[6] Ji Y, Zhang P, Xing Y, Jia L, Zhang Y, Jia T, et al. Effect of 1α, 25-dihydroxyvitamin D3 on the osteogenic differentiation of human periodontal ligament stem cells and the underlying regulatory mechanism. Int J Mol Med. 2019 Jan;43(1):167–76. doi: 10.3892/ijmm.2018.3947 30365053 PMC6257868

[7] Gambardella A, Nagaraju CK, O’Shea PJ, Mohanty ST, Kottam L, Pilling J, et al. Glycogen synthase kinase-3α/β inhibition promotes in vivo amplification of endogenous mesenchymal progenitors with osteogenic and adipogenic potential and their differentiation to the osteogenic lineage. J Bone Miner Res. 2011;26(4):811–21. doi: 10.1002/jbmr.266 20939016

[8] Gilmour PS, O’Shea PJ, Fagura M, Pilling JE, Sanganee H, Wada H, et al. Human stem cell osteoblastogenesis mediated by novel glycogen synthase kinase 3 inhibitors induces bone formation and a unique bone turnover biomarker profile in rats. Toxicol Appl Pharmacol. 2013;272(2):399–407. doi: 10.1016/j.taap.2013.07.001 23872097

[9] Wang Z, Xie Q, Yu Z, Zhou H, Huang Y, Bi X, et al. A regulatory loop containing miR-26a, GSK3β and C/EBPα regulates the osteogenesis of human adipose-derived mesenchymal stem cells. Sci Rep. 2015;5:15280. doi: 10.1038/srep15280 26469406 PMC4606799

[10] Hu B, Weng Y, Xia X-H, Liang X-J, Huang Y. Clinical advances of siRNA therapeutics. J Gene Med. 2019;21(7):e3097. doi: 10.1002/jgm.3097 31069898

[11] Saw PE, Song E-W. siRNA therapeutics: a clinical reality. Sci China Life Sci. 2020;63(4):485–500. doi: 10.1007/s11427-018-9438-y 31054052

[12] Bertrand J-R, Pottier M, Vekris A, Opolon P, Maksimenko A, Malvy C. Comparison of antisense oligonucleotides and siRNAs in cell culture and in vivo. Biochem Biophys Res Commun. 2002;296(4):1000–4. doi: 10.1016/s0006-291x(02)02013-2 12200148

[13] Wang LL, Burdick JA. Engineered hydrogels for local and sustained delivery of RNA-interference therapies. Adv Healthc Mater. 2017;6(1):10. doi: 10.1002/adhm.201601041 PMC5226889 27976524

[14] Chernikov IV, Vlassov VV, Chernolovskaya EL. Current Development of siRNA Bioconjugates: From Research to the Clinic. Front Pharmacol. 2019;10:444. doi: 10.3389/fphar.2019.00444 31105570 PMC6498891

[15] Ramamoorth M, Narvekar A. Non viral vectors in gene therapy- an overview. J Clin Diagn Res. 2015;9(1):GE01-6. doi: 10.7860/JCDR/2015/10443.5394 25738007 PMC4347098

[16] Zhu L, Mahato RI. Lipid and polymeric carrier-mediated nucleic acid delivery. Expert Opin Drug Deliv. 2010;7(10):1209–26. doi: 10.1517/17425247.2010.513969 20836625 PMC2945687

[17] Yoo JW, Hong SW, Kim S, Lee D. Inflammatory cytokine induction by siRNAs is cell type- and transfection reagent-specific. Biochem Biophys Res Commun. 2006;347(4):1053–8. doi: 10.1016/j.bbrc.2006.07.001 16857170

[18] Yang HY, Vonk LA, Licht R, van Boxtel AM, Bekkers JE, Kragten AH, et al. Cell type and transfection reagent-dependent effects on viability, cell content, cell cycle and inflammation of RNAi in human primary mesenchymal cells. Eur J Pharm Sci. 2014;:35–44. doi: 10.1016/j.ejps.2013.12.006 24345796

[19] Kozhevnikova MN, Mikaelian AS, Starostin VI. Molecular and genetic regulation of osteogenic differentiation of mesenchymal stromal cells. Izv Akad Nauk Ser Biol. 2008;(3):261–71. 18668714

[20] Takahashi-Yanaga F. Activator or inhibitor? GSK-3 as a new drug target. Biochem Pharmacol. 2013;86(2):191–9. doi: 10.1016/j.bcp.2013.04.022 23643839

[21] Lal H, Ahmad F, Woodgett J, Force T. The GSK-3 family as therapeutic target for myocardial diseases. Circ Res. 2015;116(1):138–49. doi: 10.1161/CIRCRESAHA.116.303613 25552693 PMC4283216

[22] Embi N, Rylatt DB, Cohen P. Glycogen synthase kinase-3 from rabbit skeletal muscle. Separation from cyclic-AMP-dependent protein kinase and phosphorylase kinase. Eur J Biochem. 1980;107(2):519–27. 6249596

[23] Fuentealba LC, Eivers E, Ikeda A, Hurtado C, Kuroda H, Pera EM, et al. Integrating patterning signals: Wnt/GSK3 regulates the duration of the BMP/Smad1 signal. Cell. 2007;131(5):980–93. doi: 10.1016/j.cell.2007.09.027 18045539 PMC2200633

[24] Gao C, Xiao G, Hu J. Regulation of Wnt/β-catenin signaling by posttranslational modifications. Cell Biosci. 2014;4(1):13. doi: 10.1186/2045-3701-4-13 24594309 PMC3977945

[25] Kugimiya F, Kawaguchi H, Ohba S, Kawamura N, Hirata M, Chikuda H, et al. GSK-3beta controls osteogenesis through regulating Runx2 activity. PLoS One. 2007;2(9):e837. doi: 10.1371/journal.pone.0000837 17786208 PMC1950686

[26] Shimoyama A, Wada M, Ikeda F, Hata K, Matsubara T, Nifuji A, et al. Ihh/Gli2 signaling promotes osteoblast differentiation by regulating Runx2 expression and function. Mol Biol Cell. 2007;18(7):2411–8. doi: 10.1091/mbc.e06-08-0743 17442891 PMC1924839

[27] Amano K, Densmore M, Nishimura R, Lanske B. Indian hedgehog signaling regulates transcription and expression of collagen type X via Runx2/Smads interactions. J Biol Chem. 2014;289(36):24898–910. doi: 10.1074/jbc.M114.570507 25028519 PMC4155658

[28] Grafe I, Alexander S, Peterson JR, Snider TN, Levi B, Lee B, et al. TGF-β Family Signaling in Mesenchymal Differentiation. Cold Spring Harb Perspect Biol. 2018;10(5):a022202. doi: 10.1101/cshperspect.a022202 28507020 PMC5932590

[29] Arioka M, Takahashi-Yanaga F, Sasaki M, Yoshihara T, Morimoto S, Hirata M, et al. Acceleration of bone regeneration by local application of lithium: Wnt signal-mediated osteoblastogenesis and Wnt signal-independent suppression of osteoclastogenesis. Biochem Pharmacol. 2014;90(4):397–405. doi: 10.1016/j.bcp.2014.06.011 24955980

[30] Gillespie JR, Bush JR, Bell GI, Aubrey LA, Dupuis H, Ferron M, et al. GSK-3β function in bone regulates skeletal development, whole-body metabolism, and male life span. Endocrinology. 2013;154(10):3702–18. doi: 10.1210/en.2013-1155 23904355 PMC5053811

[31] Huh J-E, Ko R, Jung HJ, Lee SY. Glycogen synthase kinase 3β promotes osteogenic differentiation of murine adipose-derived stromal cells. PLoS One. 2013;8(1):e54551. doi: 10.1371/journal.pone.0054551 23342170 PMC3546989

[32] Guo S, Zhang M, Huang Y. Three “E” challenges for siRNA drug development. Trends Mol Med. 2024;30(1):13–24. doi: 10.1016/j.molmed.2023.10.005 37951790

[33] Kim T-H, Singh RK, Kang MS, Kim J-H, Kim H-W. Inhibition of osteoclastogenesis through siRNA delivery with tunable mesoporous bioactive nanocarriers. Acta Biomater. 2016;29:352–64. doi: 10.1016/j.actbio.2015.09.035 26432439

[34] Liang M-H, Chuang D-M. Differential roles of glycogen synthase kinase-3 isoforms in the regulation of transcriptional activation. J Biol Chem. 2006;281(41):30479–84. doi: 10.1074/jbc.M607468200 16912034

[35] Liang MH, Chuang DM. Regulation and function of glycogen synthase kinase-3 isoforms in neuronal survival. J Biol Chem. 2007;282(6):3904–17. doi: 10.1074/jbc.M605178200 17148450

[36] Krivosheeva IA, Vyakhireva Y u V, Tabakov VY u, Skoblov MY u. Development of an approach to knockdown of DUX4 gene by siRNA in myoblasts derived from patients with FSHD. Medical genetics. 2021;20(3):47–56. doi: 10.25557/2073-7998.2021.03.47-56

[37] Laganà A, Veneziano D, Russo F, Pulvirenti A, Giugno R, Croce CM, et al. Computational design of artificial RNA molecules for gene regulation. Methods Mol Biol. 2015;1269:393–412. doi: 10.1007/978-1-4939-2291-8_25 25577393 PMC4425273

[38] Horova V, Hradilova N, Jelinkova I, Koc M, Svadlenka J, Brazina J, et al. Inhibition of vacuolar ATPase attenuates the TRAIL-induced activation of caspase-8 and modulates the trafficking of TRAIL receptosomes. FEBS J. 2013;280(14):3436–50. doi: 10.1111/febs.12347 23678861

[39] Vyakhireva JV, Filatova AYu, Krivosheeva IA, Skoblov MYu. siRNA-mediated gene silencing. Bulletin of RSMU. 2017;(3):17–29. doi: 10.24075/brsmu.2017-03-02

[40] Chomczynski P, Sacchi N. Single-step method of RNA isolation by acid guanidinium thiocyanate-phenol-chloroform extraction. Anal Biochem. 1987;162(1):156–9. doi: 10.1006/abio.1987.9999 2440339

[41] Mosmann T. Rapid colorimetric assay for cellular growth and survival: application to proliferation and cytotoxicity assays. J Immunol Methods. 1983;65(1–2):55–63. doi: 10.1016/0022-1759(83)90303-4 6606682

[42] Tafer H. Bioinformatics of siRNA design. Methods Mol Biol. 2014;1097:477–90. doi: 10.1007/978-1-62703-709-9_22 24639173

[43] Chaudhary A, Srivastava S, Garg S. Development of a software tool and criteria evaluation for efficient design of small interfering RNA. Biochem Biophys Res Commun. 2011;404(1):313–20. doi: 10.1016/j.bbrc.2010.11.114 21145307

[44] Takasaki S. Selecting effective siRNA target sequences by using Bayes’ theorem. Comput Biol Chem. 2009;33(5):368–72. doi: 10.1016/j.compbiolchem.2009.07.009 19682951

[45] Takasaki S. Methods for selecting effective siRNA target sequences using a variety of statistical and analytical techniques. Methods Mol Biol. 2013;942:17–55. doi: 10.1007/978-1-62703-119-6_2 23027044

[46] Abugessaisa I, Shimoji H, Sahin S, Kondo A, Harshbarger J, Lizio M, et al. FANTOM5 transcriptome catalog of cellular states based on Semantic MediaWiki. Database. 2016;2016:baw105. doi: 10.1093/database/baw105 PMC4940433 27402679

[47] Thomas P, Smart TG. HEK293 cell line: a vehicle for the expression of recombinant proteins. J Pharmacol Toxicol Methods. 2005;51(3):187–200. doi: 10.1016/j.vascn.2004.08.014 15862464

[48] Biological evaluation of medical devices. Part 5: Tests for in vitro cytotoxicity. International Organization for Standardization (ISO). 2009.

[49] Benoit DSW, Boutin ME. Controlling mesenchymal stem cell gene expression using polymer-mediated delivery of siRNA. Biomacromolecules. 2012;13(11):3841–9. doi: 10.1021/bm301294n 23020123 PMC3501203

[50] Ren Y, Gong W, Xu Q, Zheng X, Lin D, Wang Y, et al. siRecords: an extensive database of mammalian siRNAs with efficacy ratings. Bioinformatics. 2006;22(8):1027–8. doi: 10.1093/bioinformatics/btl026 16443930

[51] Pascut D, Bedogni G, Tiribelli C. Silencing efficacy prediction: a retrospective study on target mRNA features. Biosci Rep. 2015;35(2):e00185. doi: 10.1042/BSR20140147 25702798 PMC4381284

[52] Vert J-P, Foveau N, Lajaunie C, Vandenbrouck Y. An accurate and interpretable model for siRNA efficacy prediction. BMC Bioinformatics. 2006;7:520. doi: 10.1186/1471-2105-7-520 17137497 PMC1698581

[53] Truss M, Swat M, Kielbasa SM, Schäfer R, Herzel H, Hagemeier C. HuSiDa--the human siRNA database: an open-access database for published functional siRNA sequences and technical details of efficient transfer into recipient cells. Nucleic Acids Res. 2005;33(Database issue):D108-11. doi: 10.1093/nar/gki131 15608157 PMC540085

[54] Lv H, Zhang S, Wang B, Cui S, Yan J. Toxicity of cationic lipids and cationic polymers in gene delivery. J Control Release. 2006;114(1):100–9. doi: 10.1016/j.jconrel.2006.04.014 16831482

[55] Wei X, Shao B, He Z, Ye T, Luo M, Sang Y, et al. Cationic nanocarriers induce cell necrosis through impairment of Na(+)/K(+)-ATPase and cause subsequent inflammatory response. Cell Res. 2015;25(2):237–53. doi: 10.1038/cr.2015.9 25613571 PMC4650577

[56] Kafil V, Omidi Y. Cytotoxic impacts of linear and branched polyethylenimine nanostructures in a431 cells. Bioimpacts. 2011;1(1):23–30. doi: 10.5681/bi.2011.004 23678404 PMC3648946

[57] Godbey WT, Wu KK, Mikos AG. Poly(ethylenimine)-mediated gene delivery affects endothelial cell function and viability. Biomaterials. 2001;22(5):471–80. doi: 10.1016/s0142-9612(00)00203-9 11214758

[58] Hoare M, Greiser U, Schu S, Mashayekhi K, Aydogan E, Murphy M, et al. Enhanced lipoplex-mediated gene expression in mesenchymal stem cells using reiterated nuclear localization sequence peptides. J Gene Med. 2010;12(2):207–18. doi: 10.1002/jgm.1426 20082426

[59] Loiselle AE, Lloyd SAJ, Paul EM, Lewis GS, Donahue HJ. Inhibition of GSK-3β rescues the impairments in bone formation and mechanical properties associated with fracture healing in osteoblast selective connexin 43 deficient mice. PLoS One. 2013;8(11):e81399. doi: 10.1371/journal.pone.0081399 24260576 PMC3832658

[60] Sisask G, Marsell R, Sundgren-Andersson A, Larsson S, Nilsson O, Ljunggren O, et al. Rats treated with AZD2858, a GSK3 inhibitor, heal fractures rapidly without endochondral bone formation. Bone. 2013;54(1):126–32. doi: 10.1016/j.bone.2013.01.019 23337038

[61] Cook DA, Fellgett SW, Pownall ME, O’Shea PJ, Genever PG. Wnt-dependent osteogenic commitment of bone marrow stromal cells using a novel GSK3β inhibitor. Stem Cell Res. 2014;12(2):415–27. doi: 10.1016/j.scr.2013.10.002 24382458

[62] Nantavisai S, Pisitkun T, Osathanon T, Pavasant P, Kalpravidh C, Dhitavat S, et al. Systems biology analysis of osteogenic differentiation behavior by canine mesenchymal stem cells derived from bone marrow and dental pulp. Sci Rep. 2020;10(1):20703. doi: 10.1038/s41598-020-77656-0 33244029 PMC7692528

